# North American domestic pigs are susceptible to experimental infection with Japanese encephalitis virus

**DOI:** 10.1038/s41598-018-26208-8

**Published:** 2018-05-21

**Authors:** So Lee Park, Yan-Jang S. Huang, Amy C. Lyons, Victoria B. Ayers, Susan M. Hettenbach, D. Scott McVey, Kenneth R. Burton, Stephen Higgs, Dana L. Vanlandingham

**Affiliations:** 1 Department of Diagnostic Medicine and Pathobiology, College of Veterinary Medicine, Kansas State University, Manhattan, KS USA; 2 Biosecurity Research Institute, Kansas State University, Manhattan, KS USA; 3 Arthropod-Borne Animal Diseases Research Unit, Center for Grain and Animal Health Research, Agricultural Research Service, United States Department of Agriculture, Manhattan, KS USA; 4 National Agricultural Biosecurity Center, Kansas State University, Manhattan, KS USA

## Abstract

Japanese encephalitis virus (JEV) is a mosquito-borne flavivirus that is capable of causing encephalitic diseases in children. While humans can succumb to severe disease, the transmission cycle is maintained by viremic birds and pigs in endemic regions. Although JEV is regarded as a significant threat to the United States (U.S.), the susceptibility of domestic swine to JEV infection has not been evaluated. In this study, domestic pigs from North America were intravenously challenged with JEV to characterize the pathological outcomes. Systemic infection followed by the development of neutralizing antibodies were observed in all challenged animals. While most clinical signs were limited to nonspecific symptoms, virus dissemination and neuroinvasion was observed at the acute phase of infection. Detection of infectious viruses in nasal secretions suggest infected animals are likely to promote the vector-free transmission of JEV. Viral RNA present in tonsils at 28 days post infection demonstrates the likelihood of persistent infection. In summary, our findings indicate that domestic pigs can potentially become amplification hosts in the event of an introduction of JEV into the U.S. Vector-free transmission to immunologically naïve vertebrate hosts is also likely through nasal shedding of infectious viruses.

## Introduction

Japanese encephalitis virus (JEV) is a mosquito-borne flavivirus that has high human and veterinary public health significance. In the endemic Asia-Pacific region, enzootic transmission cycles of JEV are maintained among amplification hosts such as swine and avian species. Whilst the majority of infections in immunologically naïve humans lead to mild clinical symptoms such as headache, high fever, and lethargy, it is estimated that 1 in 250 infections can lead to severe neurological diseases such as paralysis, motor and memory deficits, and seizures with up to 40% case mortality rate^1–5^. Severe infections of the central nervous system can also be observed in other incidental hosts such as equid species^6,7^. On the other hand, outcomes of JEV infections in swine species are often age-dependent. While infected young piglets develop nonsuppurative encephalitis, JEV infection in mature adult pigs primarily manifests as a reproductive disease such as abortions and transient infertility, which can become important agricultural and food security issues^4,8–10^. JEV outbreaks can therefore significantly impact and threaten the agricultural economy and public health, particularly in regions where vaccine coverage is low and diagnostic capacity is limited.

Currently, the endemic region of JEV is mainly restricted in the Asia-Pacific region, covering from Southeastern Russia to Japan, Eastern China, Southeastern Asia, India, and Northern Australia, where an estimated 68,000 JEV cases occur each year^4,11^. A recent report of a viremic individual dually infected with JEV and yellow fever virus in Africa indicates the potential change in the geographic distribution of JEV and highlights the threat of expansion to new regions where amplification host species and competent vectors are present^12^. Previous studies have identified competent mosquitoes and susceptible avian species in North America that can sustain the enzootic transmission of JEV, designating the pathogen as a significant health threat^13–15^. However, the susceptibility of North American swine to JEV infection and its disease outcomes remains largely unknown despite the significant role that pigs play in JEV-endemic regions as efficient amplifying hosts^4,16^. Results from JEV challenge experiments in pigs derived from endemic regions fail to directly demonstrate the degree of susceptibility to JEV among domestic pig breeds that are used in North America for pork production. Additionally, the majority of published studies were performed with JEV strains belonging to genotype III (GIII), which was previously dominant in the endemic region but has been replaced by the rapidly emerging strains under the clade b of genotype I (GI-b)^10,17–20^. Such a critical gap of knowledge precludes the comprehensive assessment needed to estimate the risk and develop effective countermeasures against the potential emergence of JEV in the United States (U.S.).

In this study, the common North American white-line crossbreed of domestic pigs were challenged with a GI-b JEV strain to determine their susceptibility to the newly emerging genotype of JEV. Pathogenic outcomes and tissue tropism were characterized by detection of infectious viruses and viral genomes.

## Results

### Clinical outcomes, viremic profile, and seroconversion of animals challenged with JEV

The animals were healthy and seronegative to JEV prior to the experimental challenge. Fever, weight loss, depression, lethargy, and hind limb ataxia were observed in JEV-infected pigs after inoculation, but most clinical signs resolved within one week. High fevers up to 41 °C were observed in infected pigs as early as day 1 post-infection (PI) and lasted four to five days before temperatures decreased to within normal limits (<40 °C) (Supplementary Fig. S1a). Although not statistically significant, minor weight loss was recorded in 50% (5/10, *p* = 0.454) of infected pigs between 1 and 2 day post-infection (DPI) and in all infected pigs (10/10, *p* = 0.635) between 3 and 4 DPI (Supplementary Fig. S1b). Challenged pigs also exhibited depression and lethargy since 1 DPI. While all returned to bright, alert, and responsive states after 5 DPI, one pig continued to be slightly depressed until 13 DPI. The same animal also developed a second fever peak of 40.4 °C on day 8 PI that resolved in three days (Supplementary Fig. S1a). Mild ataxia in the rear legs was then subsequently observed between 10 to 13 DPI. Two other infected pigs also exhibited gait abnormalities of the rear legs on 19 DPI that resolved by 27 DPI.

**Figure 1:**
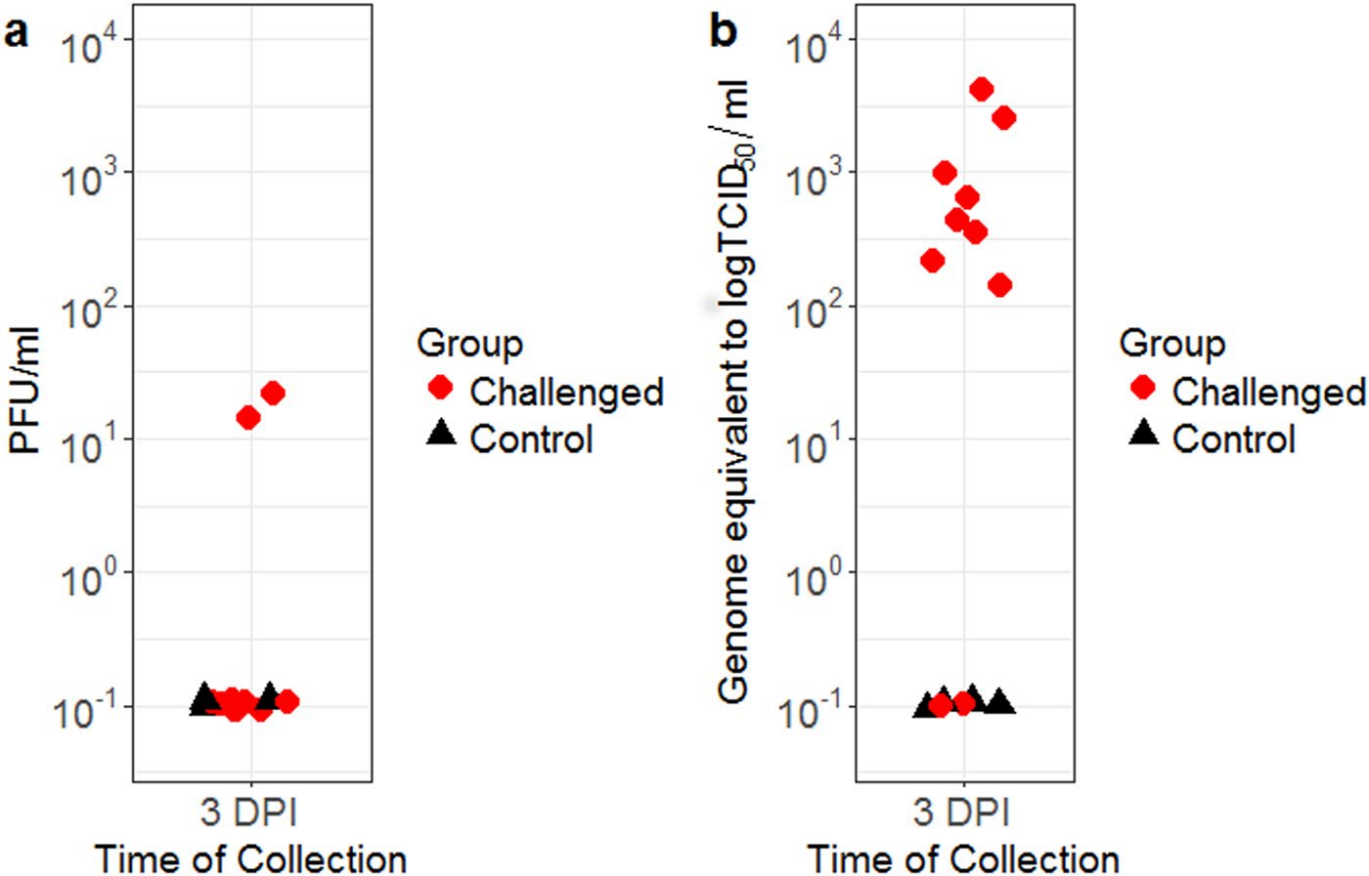
Viral titers of serum collected at day 3 following JEV challenge quantified by plaque assay (**a**) and RT-qPCR (**b**). PFU = plaque forming units. DPI = day post-infection.

Viremia was detected in all of the infected animals tested. Figure 1 summarizes the viral titers of serum samples collected at 3 DPI. Serum samples from two infected pigs showed detectable levels of infectious viruses by plaque assay, reaching up to 2.0 × 10^1^ plaque forming units (PFU)/ml (Fig. 1a). However, quantitative reverse-transcriptase polymerase chain reaction (RT-qPCR) demonstrated that at least eight animals developed viremia at 3 DPI with viral RNA loads ranging between 1.34 × 10^2^ and 4.2 × 10^3^ genome equivalent-50% tissue culture infectious dose (geq-TCID_50_)/ml (Fig. 1b). Viremia titer of 6.07 geq-TCID_50_/ml was detected in one challenged animal at 5 DPI. These results suggests that clearance of viremia in domestic pigs can take place as early as three days after intravenous challenge. The recovery from the acute phase of infection was also demonstrated as all animals developed neutralizing antibodies at a geometric mean titer of 1:243 at 28 DPI.

### Viral shedding in nasal secretions

**Figure 2:**
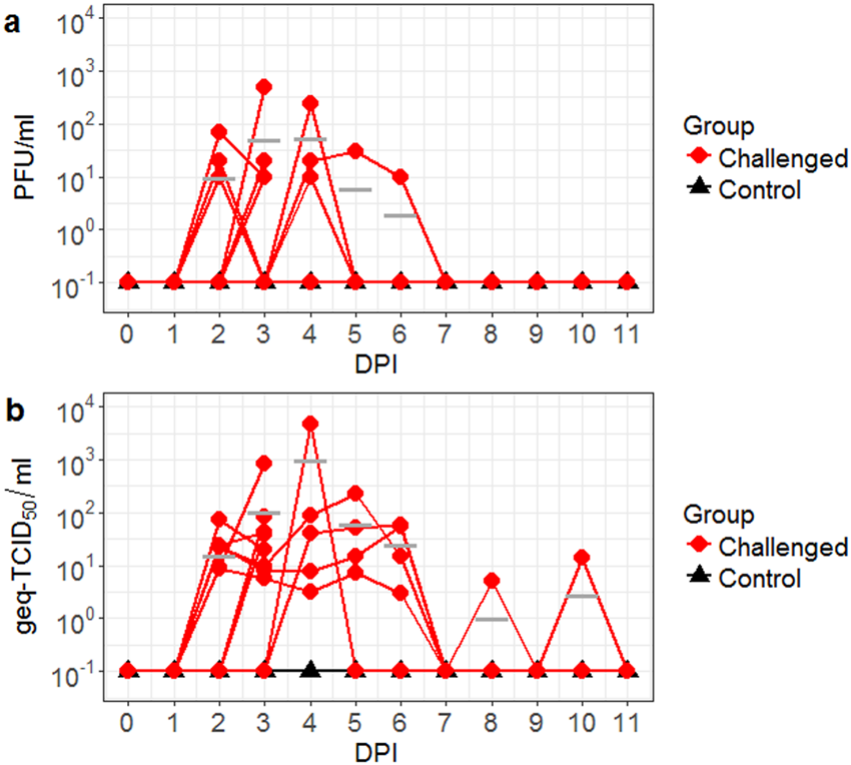
Nasal shedding of JEV by experimentally infected pigs quantified by plaque assay (**a**) and RT-qPCR (**b**). PFU = plaque forming units. DPI = day post-infection. Geq-TCID_50_ = genome equivalent-50% tissue culture infectious dose. Bar lines indicate the mean of the values collected from the challenged animals.

To characterize the nasal shedding dynamics of JEV in pigs, secretions from the nose were collected daily from alternating nares for virus titration. Infectious virus was detected in the nasal swab samples by 2 DPI for up to five days, as shown in Fig. 2a. Up to 90% (9/10) of the infected pigs were actively shedding infectious virus at various time points for a period of one to five days. At 3 DPI, the highest infectious titer was observed at 4.8 × 10^2^ PFU/ml. While viremia was cleared prior to 5 DPI, 60% (3/5) of infected pigs continued to shed viruses to 4 DPI. Shedding of infectious virus persisted for up to six days in an infected pig. By 7 DPI, no nasal swabs were positive for JEV.

Similar shedding kinetics were observed via RT-qPCR (Fig. 2b). JEV shedding was detectable at 2 DPI, at average titers of 1.6 × 10^1^ geq-TCID_50_/ml. At 3 DPI, 80% (8/10) of the challenged pigs shed between 5.62 and 8.18 × 10^2^ geq-TCID_50_/ml. A peak titer of 4.76 × 10^4^ geq-TCID_50_/ml was detected at 4 DPI. While most challenged animals stopped shedding after 6 DPI, one animal continued to shed between 0 and 1.4 × 10^1^ geq-TCID_50_/ml until 10 DPI.

### Detection of infectious viruses and viral genome in tissues collected at the acute phase of infection

**Figure 3:**
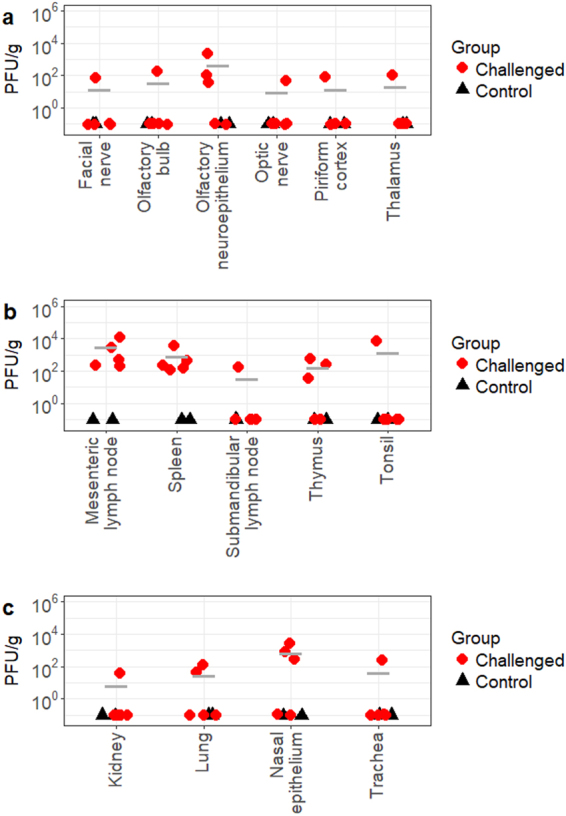
Infectious viral titers of JEV-positive CNS (**a**), lymphoid (**b**), and other (**c**) tissues collected at 3 DPI. PFU = plaque forming units. Bar lines indicate the mean of the values collected from the challenged animals.

The dissemination and tissue tropism of JEV at the acute phase of infection were determined by the titration of tissue samples harvested at 3 DPI. The presence of infectious virus demonstrated that infection of JEV can lead to neuroinvasion among North American domestic pigs. As shown in Fig. 3a, infectious virus was recovered from six nervous tissue samples (facial nerve, olfactory bulb, olfactory neuroepithlium, optic nerve, piriform cortex, and thalamus) with titers ranging from 5.0 × 10^1^ PFU/g to 1.9 × 10^2^ PFU/g. Infectious virus was present in the olfactory neuroepithelium of 60% (3/5) of infected pigs, reaching titers up to 2.1 × 10^3^ PFU/g but not statistically significantly higher (0.111 ≥ *p* ≥ 0.093) compared to other positive neural tissues (Supplementary Table S1).

As summarized in Fig. 3b, infectious virus was also detected in the lymphatic system of challenged animals, indicating the systemic spread of JEV at the acute phase of infection. Mesenteric lymph nodes and spleen of all challenged animals were positive for infectious viruses at average titers of 3.1 × 10^3^ PFU/g and 9.0 × 10^2^ PFU/g, respectively. Mesenteric lymph nodes had significantly higher viral titers (0.046 ≥ *p* ≥ 0) than other positive neural and lymphoid tissues except spleen (Supplementary Table S1). The presence of infectious virus was also observed in the tonsil of one animal at the titer of 7.3 × 10^3^ PFU/g. Dissemination of JEV was observed in other tissues including the trachea, lungs, and kidneys (Fig. 3c). Out of the positive peripheral tissues, nasal epithelium had a particularly high mean infectious viral titer of 7.53 × 10^2^ PFU/g, with a peak of 2.6 × 10^3^ PFU/g in one challenged pig but the values were not statistically significant (*p* ≥ 0.061) unless compared to kidneys (*p* = 0.043) (Supplementary Table S1). Titration of other central nervous system (CNS), lymphoid, and visceral tissues including different regions of the cerebral cortex, brainstem, cerebellum, spinal cord, Peyer’s patches, liver, skeletal muscle, and reproductive tract, failed to identify the presence of infectious viruses.

**Figure 4:**
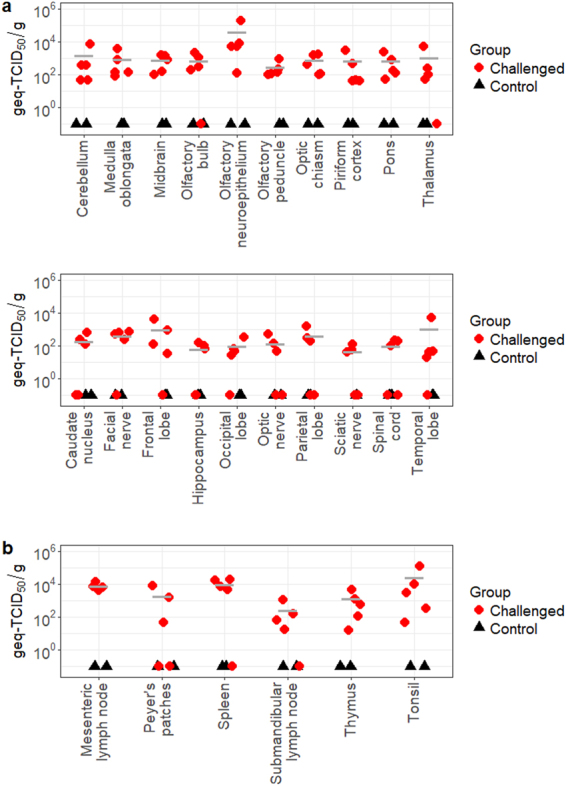
Viral load of CNS (**a**) and lymphoid (**b**) tissues collected at 3 DPI, as estimated by RT-qPCR. Geq-TCID_50_ = genome equivalent-50% tissue culture infectious dose. Bar lines indicate the mean of the values collected from the challenged animals.

The systemic infection and neuroinvasion of JEV were demonstrated by JEV-specific RT-qPCR, as depicted in Fig. 4. As expected, the RT-qPCR assay used in this study provided a higher sensitivity than plaque assay in detecting the presence of JEV. Consistent with the results of plaque assays, homogenized olfactory neuroepithelium had significantly higher viral load than most neural tissues (0.042 ≥ *p* ≥ 0.001) with the highest viral load at 1.8 × 10^6^ geq-TCID_50_/g (Fig. 4a and Supplementary Table S1). The lowest mean titer of 4.4 × 10^1^ geq-TCID_50_/g was recovered from the sciatic nerve. Other notable CNS structures with average viral loads above 10^3^ geq-TCID_50_/g included the cerebellum (1.6 × 10^3^ geq-TCID_50_/g), thalamus (1.1 × 10^3^ geq-TCID_50_/g), temporal lobe (1.1 × 10^3^ geq-TCID_50_/g), and frontal lobe (1.0 × 10^3^ geq-TCID_50_/g). However, these structures were not statistically significant unless compared to olfactory neuroepithelium (*p* = 0.042 and 0.009 for thalamus and temporal lobe, respectively).

Among the lymphoid structures of the infected pigs, the submandibular lymph nodes had the lowest viral load ranging from 1.7 × 10^1^ to 1.0 × 10^3^ geq-TCID_50_/g (Fig. 4b). Similar to the infectious viral titer results, tonsils, mesenteric lymph nodes, and the spleen produced the highest average viral RNA titers of 2.6 × 10^4^ geq-TCID_50_/g, 7.6 × 10^3^ geq-TCID_50_/g, and 9.7 × 10^3^ geq-TCID_50_/g, respectively. They were also statistically significantly higher than those of the submandibular lymph nodes (0.003 ≥ *p* ≥ 0.046) (Supplementary Table S1). The same infected pig that produced the highest viral load in the olfactory neuroepithlieum also produced the highest viral load in the tonsils, which reached 1.1 × 10^5^ geq-TCID_50_/g. In summary, multiple nervous and lymphatic tissues showed the positive detection of viral RNA by RT-qPCR. These results demonstrate the high incidence of neuroinvasion and systemic infection among the animals challenged with JEV.

### Viral clearance and persistent infection of JEV

Titration of homogenized tissues collected from the five challenged animals at 28 DPI failed to detect any infectious viruses. However, viral genome was detected by RT-qPCR in the tonsils. Viral loads of tonsils collected from three infected pigs ranged between 4.9 × 10^1^ to 3.4 × 10^2^ geq-TCID_50_/g, indicating that there was active ongoing viral replication occurring in this structure although no live viruses could be isolated by plaque assay.

## Discussion

Our results demonstrate that North American domestic pigs, as used for commercial pork production, are susceptible to JEV infection. North American pigs infected with GI-b JEV JE-91 strain developed clinical signs including depression, fever, and minor weight loss followed by mild to moderate bilateral hind limb ataxia, which is a clinical finding often reported with other ambulatory abnormalities in horses infected with JEV^6,21^. Previous experimental challenge studies with pigs from Asia and Europe inoculated with GIII virus strains reported similar clinical signs^9,10,22,23^, although neurologic signs such as hind limb tremors were only documented in the studies conducted in Japan^10,23^. This suggests that JEV susceptibility between pigs from different regions and/or pathogenicity of GIII and GI-b may not be significantly different from each other. Nonetheless, a JEV outbreak in the pig population in North America could potentially be as or more detrimental as those reported in the endemic regions due to the lack of JEV-specific maternal antibodies and herd immunity to the foreign virus^24^.

Clinical disease was also coupled with viremia and viral shedding. Viral titers as high as 4.2 × 10^3^ geq-TCID_50_/ml were detected at 3 DPI in the serum of infected North American pigs (Fig. 1b). This amount of virus in the blood is slightly lower compared to previous reports, in which higher than 10^4^ infectious virus quantities per ml were reported^22,25–27^. A possible explanation may be the day of collection as viremia in European pigs after needle JEV challenge reached a peak of approximately 10^4^ RNA units equivalent to TCID_50_/ml (U/ml) at 1 DPI and became as low as 10^1^ U/ml at 3 DPI^22,25^. Nonetheless, our reported viremias based on genome equivalent data may be sufficient to infect feeding mosquitoes^4,28^. Albeit the low infection rates, infection of *Culex tritaeniorhynchus* could be established at infectious titers as low as 10^1.5^ LD_50_^28,29^. The event of subsequent transmissions to other susceptible vertebrate hosts is possible in the presence of highly susceptible mosquito species. In the meantime, transmission in the absence of competent vectors may potentially also occur between susceptible vertebrates based on recent findings on the significance of nasal shedding of JEV^25^. While most infected pigs in this study shed an average viral titer of 7.2 × 10^1^ PFU/ml or 2.25 × 10^2^ geq-TCID_50_/ml in the nasal secretions (Fig. 2), Ricklin *et al*.^25^ demonstrated that viral titers as low as 10 TCID_50_/ml can be infectious to pigs via the intranasal route. Therefore, once a pig becomes infected, animal-to-animal transmission may occur throughout the entire herd. The risk for vector-free aerosol or contact transmission of JEV from pigs to humans is currently unknown, but intranasal infection of JEV has been demonstrated in other vertebrate species including rhesus monkeys^29^, macaques^30,31^, and mice^32,33^.

In terms of tissue tropism and virus dissemination, JEV behaved similarly as reported in previous published studies^8,22,34,35^ and displayed tropism for nervous and lymphoid tissues in North American pigs. Titration and quantification of viral RNA via RT-qPCR of homogenized tissue samples identified the following tissues with the highest viral titers at 3 DPI: nasal epithelium, olfactory neuroepithelium, mesenteric lymph node, spleen, and tonsil. The highest values from these structures ranged from 2.1 × 10^3^ PFU/g to 1.2 × 10^4^ PFU/g or 3.5 × 10^3^ geq-TCID_50_/g to 3.6 × 10^4^ geq-TCID_50_/g (Figs 3 and 4). Such high titers at the acute stage of infection, particularly in the nasal epithelium and olfactory epithelium, highlight two significant findings. Firstly, the source of nasal shedding may be attributed to virus replicating in either the nasal epithelium or olfactory neuroepithelium, although JEV antigens could not be detected in these structures in Asian pigs after JEV intranasal challenge in a previous study^23^. Secondly, the high viral titers detected in the olfactory neuroepithelium at the acute stage of infection provides support to the previous finding that JEV can reach the brain through the olfactory pathway in pigs^23^. Similarly to other encephalitic alphaviruses such as Sindbis^36^ and Venezuelan equine encephalitis^37^ and encephalitic flaviviruses such as St. Louis encephalitis^38^ and Murray Valley encephalitis viruses^39^, JEV can bypass the blood-brain-barrier to reach the brain by retrograde axonal transport through the olfactory neuroepithelium in addition to the hematogenous route of brain infection described in other studies^10,40^. Since JEV neuroinvasion is regarded to be age-dependent^40–42^, it would be interesting to investigate if this pattern of viral infection and dissemination is also observed in adult pigs, which reportedly only experience reproductive disease from JEV infection^4,7^.

Another significant finding in this study was viral persistence in the tonsils. While no infectious virions could be isolated, viral RNA loads approximately 10^1^ to 10^2^ geq-TCID_50_/g were detected at 28 DPI in the tonsils of infected North American pigs. This discrepancy may have occurred because the amount of live infectious viruses in the tonsil were below the limit of detection of cell-based detection methods like plaque assay. Nonetheless, comparable results were also observed in an European study, in which upwards of 10^4^ RNA units equivalent to TCID_50_/g of JEV were detected at 25 DPI in the tonsils of their local domestic pigs after needle-challenge^25^. This is an important finding, because this may indicate that pigs could remain as potential carriers for at least a month after the initial infection, further emphasizing the significant role that pigs play in JEV transmission. Whether or not this “silent” infection can lead to the reactivation of viremia or nasal shedding later on remains undetermined. However, other animal viruses that persistently infect tonsils, such as Bovine herpesvirus 1^43^ and Porcine reproductive and respiratory syndrome virus^44,45^, have been documented to be able to reactivate and cause secondary infections. Therefore, persistent infection of JEV in pigs warrants further investigation as it can have potential significant implications to disease transmission and control.

It is also important to interpret the results of this study with caution. First, the present study demonstrated that juvenile pigs in North America are susceptible to JEV. While young piglets of the common domestic white-line crossbreed were used as representative pigs of North America, the observations may not be directly extrapolated to the disease pathogenesis of JEV in adult pigs. However, their susceptibility to the foreign virus remains important and relevant as there are continuously stable populations of young piglets available due to the high turnover rate of pigs in swine and pork production. Second, although intravenous injection does not mimic the natural route of transmission, it allows the comparison of susceptibility and infection outcomes between domestic pigs in North America and other regions as other challenge experiments have used similar approaches^10,23,25^. Moreover, Ricklin *et al*.^25^ reported that the different modes of infection used in their study (intradermal/intravenous combination and intranasal) did not result in fundamental differences in CNS lesions or tropism and level of neutralizing antibody titers.

Collectively, our study demonstrates for the first time that North American domestic pigs can contribute to the JEV transmission cycle as amplifying hosts. Along with the recent evaluations of North American mosquitoes^13,15^ and avian species to JEV infection^14^, the present study further highlights that there are competent mosquito vectors and susceptible amplifying hosts present in North America that can support and maintain JEV transmission. As such, JEV may have the potential to become endemic in the U.S. after an introductory event similar to the recent emergence of West Nile virus, a closely related flavivirus^46^. With this potential risk, it is important to continue the international surveillance of JEV and possibly also locally in the U.S. by implementing JEV diagnostic methods, such as antibody or viral RNA detection, into the standard work up for quick identification and response as JEV is both a significant swine and human pathogen that cannot be ignored.

## Materials and Methods

### Virus and cell lines

JEV strain JE-91, an isolate under GI-b, was propagated in *Aedes albopictus* C6/36 cells maintained in Leibovitz (L-15) media, harvested, and stored at −80 °C until use, as previously reported by Huang *et al*.^15^. The strain was originally isolated from mosquitoes collected in Korea in 1991^47^. Prior to the experiment, the virus has been passaged once in Vero cells and once in C6/36 cells. The nucleotide and amino acid sequences of its envelope protein has been previous determined (GenBank access number: GQ415355). Infectious virus titers of the stocks were determined by TCID_50_ method with African green monkey kidney Vero76 cells maintained in L-15 media, as previously described^48^.

### Animal experiment and design

The following experimental procedures and animal use were approved by the Kansas State University (K-State) Institutional Animal Care and Use Committee. All methods were carried out in accordance with the approved protocol and relevant regulations. Fourteen three-week-old U.S. commercial pigs (white-line crossbreed) were housed in biosafety level 3 agriculture (BSL3-Ag) conditions at the Biosecurity Research Institute at K-State, Manhattan, KS. After five days of acclimation, the pigs were inoculated intravenously (IV) with either 1 ml of JE-91 JEV strain at 10^7^ TCID_50_/ml (n = 10) or 1 ml of sterile saline (n = 4). Challenged and control pigs were kept in separate pens to avoid the non-vector transmission described by Ricklin *et al*.^25^. To characterize the acute and convalescent stages of infection, groups of seven pigs (five infected and two control pigs) were sacrificed at days 3 and 28 PI, respectively. At termination, the pigs were first sedated by intramuscular injection of 10–20 mg/kg of ketamine and 2–3 mg/kg of xylazine, and then euthanized with IV injection of 390 mg/ml of sodium pentobarbital.

For the duration of the study, all animals were monitored daily for any clinical signs, including fever (≥40 °C), depression, diarrhea, weight loss, gait abnormalities, and neurological signs. Whole blood samples were collected via the right external jugular vein at 0, 3, 5, 7, 14, 21, and 28 DPI. Serum samples were obtained through centrifugation of coagulated blood at 2,000 × g for 10 minutes at 4 °C. Individual nasal swab samples were obtained daily from 0 to 28 DPI from alternating nares and vortexed in 1 ml of L-15 media. At necropsy, approximately 5 mm^3^ blocks of the following tissues were collected in 1 ml of L-15 media to characterize the viral dissemination and tissue tropism of JEV: brain and spinal cord of the CNS, peripheral nerves, eye, olfactory neuroepithelium, nasal turbinates, trachea, heart, lung, small intestine, liver, spleen, kidney, lymph nodes, tonsil, and skeletal muscle. All samples were stored at −80 °C prior to all analyses.

### Detection of infectious viruses and viral genome

To avoid the interference of detection created by the cytotoxicity of serum and homogenized tissues, standard plaque assay using Vero76 cells was performed to detect infectious viruses in serum, nasal swabs suspended in culture media, and homogenized tissues, as previously described^49,50^. Tissues were weighed and homogenized using the TissueLyser II system (Qiagen) at 26 Hz for four minutes followed by centrifugation at 10,000 × g for 10 minutes. Inoculum consisting of serum, nasal swab samples, and homogenized tissues was adsorbed at 37 °C for one hour. The plaques were counted after five days of incubation at 37 °C followed by staining with 1% crystal violet solution. Titers of infectious viruses were calculated in PFU/ml or PFU/g.

Genome equivalents in serum and homogenized tissues were determined using a previously published TaqMan one-step RT-qPCR assay targeting the genomic fragment encoding the nonstructural protein 5^51^. Viral RNA was first extracted from serum with the QIAamp viral RNA extraction kit (Qiagen). Total RNA was extracted from homogenized lymphoid and CNS tissue samples collected at 3 DPI with Trizol LS (Invitrogen). To determine the persistent infection in tonsil reported by Ricklin *et al*.^25^, tonsils collected at 28 DPI were also homogenized and subjected to RNA extraction with Trizol LS. For each sample reaction, the iTaq Universal Probe One-step kit (Bio-Rad) was used to prepare 20 uL total reaction mixture containing the following: 4 uL of RNA, 10 pmol of forward primer (5′ATCTGGTGYGGYAGTCTCA3′), 10 pmol of reverse primer (5′CGCGTAGATGTTCTCAGCCC3′), 4 pmol of 5′ 6-carboxyfluorescein (FAM)-labeled probe with internal ZEN, and 3′ tetramethylrhodamine quencher (5′FAM-GGAACGCGATCCAGGGCAA-IABkFQ3′). Reactions were performed on the CFX96 Real-Time PCR Detection System (Bio-Rad) with the cycling parameters described by Pyke *et al*.^51^. For each reaction, a standard curve was generated by 10-fold serial dilution of RNA extract derived from a JEV stock of known titer at 8.52 log_10_TCID_50_/ml. Results were reported as geq-TCID_50_/ml. Samples were considered positive when the Ct value was lower than 33.

### Plaque reduction neutralization test

To determine the neutralizing antibody titers, plaque reduction neutralizing tests (PRNT) were performed following the procedures described by Roehrig *et al*.^52^. All serum samples were first heat inactivated at 56 °C for 30 minutes and dilutions between 1:10 and 1:640 were tested. Approximately 50 PFU of JE-91 strain was added to each serum concentration and incubated for 1 hour at 37 °C prior to infection of Vero76 cells in six-well plates. The wells were then gently washed with Dulbecco’s phosphate-buffered saline and overlaid with 1% methylcellulose. After five days of incubation at 37 °C, the plaques were counted and the neutralizing antibody titers were calculated based on a 50% or greater reduction in plaque counts (PRNT_50_).

### Statistical analyses

The R software (version 3.4.1, The R Foundation) was used for data graphical display. SPSS Statistics software (IBM) was used for all statistical analyses. Groups were compared with nonparametric independent-samples Mann-Whitney U test and nonparametric independent-samples Kruskal-Wallis test with significance level set at *p* < 0.05. All data generated or analyzed during this study are included in this published article.

## Electronic supplementary material

Figure S1

Table S1

Dataset 1
