# Supplementary material for: North American domestic pigs are susceptible to experimental infection with Japanese encephalitis virus
Source: Sci Rep. 2018 May 21;8:7951. doi: 10.1038/s41598-018-26208-8 (PMC5962597; doi:10.1038/s41598-018-26208-8)
Supplement: Supplementary file 1 — Figure S1 [file 41598_2018_26208_MOESM1_ESM.docx]

North American domestic pigs are susceptible to experimental infection with Japanese encephalitis virus

So Lee Park^a,b^, Yan-Jang S. Huang^a,b^, Amy C. Lyons^a,b^, Victoria B. Ayers^a,b^, Susan M. Hettenbach^b^, D. Scott McVey^a,c^, Kenneth R. Burton^b,d^, Stephen Higgs^a,b^, Dana L. Vanlandingham^a,b,*^

*^a^Department of Diagnostic Medicine and Pathobiology, College of Veterinary Medicine, Kansas State University, Manhattan, KS, USA*

*^b^Biosecurity Research Institute, Kansas State University, Manhattan, KS, USA*

*^c^Arthropod-Borne Animal Diseases Research Unit, Center for Grain and Animal Health Research, Agricultural Research Service, United States Department of Agriculture, Manhattan, KS, USA*

*^d^National Agricultural Biosecurity Center, Kansas State University, Manhattan, KS, USA*


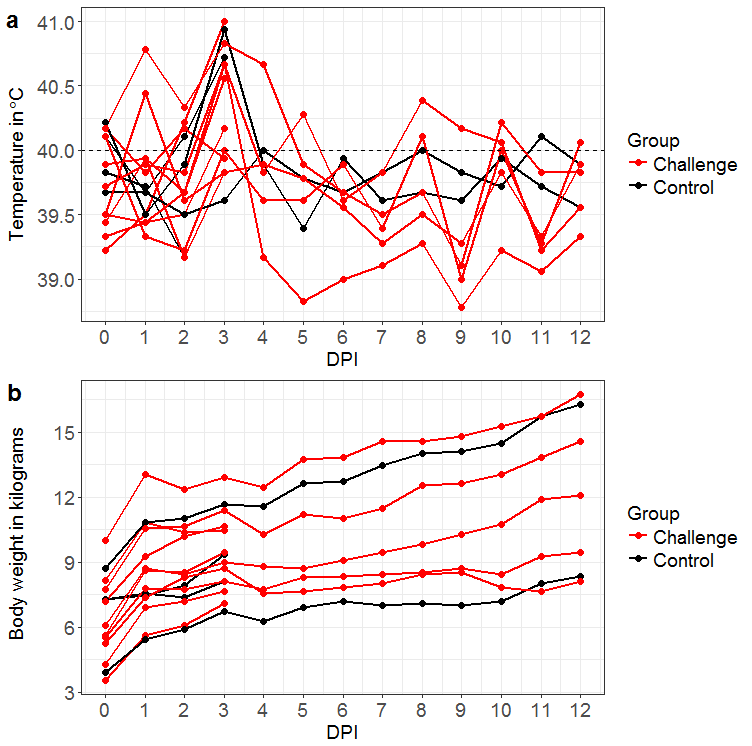


**Supplementary Figure S1.** Body temperature (a) and weight (b) of challenged and control pigs. DPI = day post-infection.
